# Supplementary material for: Syntactic denoising and multi-strategy auxiliary enhancement for aspect-based sentiment analysis
Source: PLoS One. 2025 Aug 12;20(8):e0329018. doi: 10.1371/journal.pone.0329018 (PMC12342293; doi:10.1371/journal.pone.0329018)
Supplement: Attachment — Submitted filename: Response_to_Reviewers_auresp_2.docx [file pone.0329018.s002.docx]

Response Letter

Dear editors and reviewers,

We are very grateful for your constructive comments and suggestions for our manuscript entitled “Syntactic denoising and multi-strategy auxiliary enhancement for Aspect-based Sentiment Analysis” (ID: “PONE-D-25-17433”). Your comments are very valuable and helpful for improving our manuscript. In the following, the responses to all the comments are provided one by one.

We have tried our best to make all the revisions clear, and we hope that the revised manuscript can satisfy the requirements for publication.

Sincerely,

Corresponding author.

Yunhai Zhu

Response to the comments of Editor

Editor Comment 1:Please have a dedicated section for discussion that is aligned with the research objectives.

Response :We appreciate this suggestion. In the revised manuscript, we have created a standalone section titled "Discussion" that explicitly aligns with the research objectives. This section highlights how the proposed SDMAE method addresses the two primary challenges identified in the study—syntactic noise interference and inefficient semantic-syntactic integration. We further elaborate on the performance impact of each component (AOSD and MAAM) through empirical evidence, ensuring that the discussion is directly connected to the research goals.

Editor Comment 2:Add a separate section or subsections for threats to validity, limitations, and implication of the study.

Response:Thank you for pointing this out. In response, we have reorganized and expanded the original text into a dedicated section titled “Threats to Validity and Limitations”, which now includes clearly defined subsections addressing:

Construct Validity: discussing possible annotation bias and the generalization of syntactic rules.

Internal Validity: detailing hyperparameter tuning and overfitting risks.

External Validity: clarifying the limitations in generalizing beyond English review data.

Limitations of SDMAE: covering sentiment knowledge coverage, complex sentence structure handling, and domain adaptability.

Response to the comments of Reviewer 1

Q1: The error analysis is not presented in the paper. It is recommended to present it and describe each case.

Response1:Thanks for your kind suggestion. We have added a specific case error analysis of the proposed model after the ablation experiment. Combining with the visual diagram of the AOSD algorithm can provide a more intuitive understanding of the errors.

Q2: It is not clear if hyperparameter tuning is performed. The authors have presented only one set of hyper parameters of each dataset. Having the same parameters for all the models shows that the hyper parameter tuning is not performed.

Response2: Thanks for your kind suggestion. Our experimental results are all optimal outcomes obtained through hyperparameter tuning. We provide the optimal parameter values of different models, and for the tuning process, we offer a detailed ablation experiment analysis report of the model, including the ablation experiment on the number of attention heads. The hyperparameters of the algorithm in the training process are not the key innovative part of the model.

Q3: It is recommended to present the limitations of the work.

Response3: Thanks for your kind suggestion. Thanks for your kind suggestion. We noticed that the article lacked a description of the model's limitations, and we have added an explanation of the model's limitations in the summary section of the article.

Q4: In the figure only AOSD matrix is presented. What about others? It is recommended to present and compare with others as well.

Response4: Thanks for your kind suggestion. In the ablation experiment of the AOSD algorithm, the original matrix A is the visual diagram of other models, and the matrix M is the AOSD algorithm matrix.

Q5: At the end of the related work section mentioning the uniqueness of the work is important. So it will be easier to follow and express the uniqueness properly.

Response5: Thank you for your suggestion. We have mentioned the uniqueness of this work at the end of the related work section.

Q6: The motivation of the work should be mentioned in the introduction and how they have arrived at the approach.

Response6: Thank you for your valuable suggestion. The motivation of this work has already been mentioned. It has been highlighted in red in the revised version of the introduction.

Q7: The conclusion section is not explicitly mentioned. THough the authors have presented the future work and results. It is suggested to explicitly mention the section.

Response7: Thank you for your valuable suggestion. We have proposed future work and outcomes, and clearly suggested the direction of future work.
